# The prevalence and risk factors of pressure ulcers among residents of long-term care institutions: a case study of Kazakhstan

**DOI:** 10.1038/s41598-024-57721-8

**Published:** 2024-03-26

**Authors:** Zhuldyz Zhetmekova, Laura Kassym, Assiya Kussainova, Almira Akhmetova, Irma Everink, Ainash Orazalina, Galiya Zhanaspayeva, Ainur Botabayeva, Dana Kozhakhmetova, Rauza Olzhayeva, Yuliya Semenova

**Affiliations:** 1 Nursing Department, Semey Medical University, Semey, Kazakhstan; 2 Department of General Medical Practice with a Course of Evidence-Based Medicine, Astana Medical University, Astana, Kazakhstan; 3 Department of Infectious Diseases, Dermatovenereology and Immunology, Semey Medical University, Semey, Kazakhstan; 4 Department of Health Services Research and Care and Public Health Research Institute (CAPHRI), Maastricht University, Maastricht, the Netherlands; 5 Department of Molecular Biology and Medical Genetics Named After the Academician of National Academy of Sciences Republic of Kazakhstan Raissov T. K., Semey Medical University, Semey, Kazakhstan; 6 National Scientific Center of Traumatology and Orthopedics Named After Academician Batpenov N.D., Astana, Kazakhstan; 7 Department of Internal Diseases and Rheumatology, Semey Medical University, Semey, Kazakhstan; 8 Department of Biochemistry and Chemical Disciplines Named D.M.S., Professor Tapbergenov S.O., Semey Medical University, Semey, Kazakhstan; 9 School of Medicine, Nazarbayev University, Astana, Kazakhstan

**Keywords:** Health care, Risk factors

## Abstract

Limited information is available regarding the prevalence of pressure ulcers (PUs) in residential homes in Central Asia. Therefore, the aim of this study was to identify the prevalence rates and risk factors associated with PUs among residents of long-term care medical institutions in the Republic of Kazakhstan. This cross-sectional study was conducted in four long-term care institutions in Kazakhstan. The study sample consisted of 640 patients who were assessed for the presence of PUs and associated risk factors. The evaluation was performed using the International Prevalence Measurement of Care Quality (Landelijke Prevalentiemeting Zorgkwaliteit, LPZ), the Braden scale, and the Care Dependency Score (CDS). The overall prevalence of PUs, classified as categories I–IV, was found to be 37%. When excluding category I PUs, the prevalence decreased to 35.6%. The odds ratios (ORs) for presenting with PUs were as follows: history of stroke (OR 5.22), diseases of the digestive system (OR 10.01), presence of spinal cord lesions/paraplegia (OR 20.50), recent reported confusion within the last 7 days (OR 184.00), and limited extent dependency according to the CDS (OR 4.44; 95%CI 1.31–16.1). It is imperative to establish specialized training programs aimed at equipping medical personnel, relatives, and patients themselves with the necessary skills to provide optimal care for individuals affected by PUs.

## Introduction

Pressure ulcers (PUs), also known as bedsores or pressure sores, develop as a result of exposure of the skin to either mild to moderate pressure over a long period or a significant amount of pressure over a short duration. The primary underlying factor contributing to their development is impaired blood circulation in the pressured area of the skin, leading to hypoxia and a reduced supply of nutrients. This, in turn, causes skin degradation and the formation of ulcers. PUs can range in severity, from reddened patches of skin to open wounds that extend to underlying tissues such as muscles and bones. While they are commonly observed in bony areas of the body, such as the heels, elbows, hips, and the base of the spine, PUs can appear anywhere^1^.

In acute care settings, the prevalence of PUs has been estimated to range between 6 and 18.5%^2^ despite advancements in the management of this potentially preventable condition. Some studies have reported a growing trend in the incidence of PUs^2,3^, while others have reported a declining trend^4^. Aside from pressure exerted by hard surfaces, there are several predisposing factors associated with pressure ulcer (PU) development. These include excess skin moisture, skin dehydration, and muscle spasms that expose the skin due to involuntary movements. Immobility is the primary risk factor, which can be caused by conditions such as spinal cord injury, brain damage, motor nerve disorders (e.g., Parkinson’s disease or multiple sclerosis), bone fractures, osteoarthritis, or coma states. Inadequate nutrition, particularly a deficiency in proteins, essential vitamins, and minerals, is another risk factor as it limits the skin’s ability to regenerate. Patients with both type 1 and type 2 diabetes mellitus (DM) are at an increased risk of developing PUs due to compromised immunity and associated vasculopathy, similar to patients with peripheral arterial disease (PAD). Heart and kidney failure may also contribute to the formation of PUs due to inadequate blood flow and the accumulation of toxins in the skin. Chronic obstructive pulmonary disease (COPD) has also been reported to be a provoking factor for PUs due to associated hypoventilation and hypoxia^5^.

Old age is a vulnerable period in human life characterized by an increased burden of diseases that affect mobility, compromise immunity, and impair adequate blood circulation^6^. The elderly often experience increased dependency and require care provided by others, leading to their residency in long-term care institutions. Sometimes, the occurrence of PUs is attributed to inappropriate nursing care; however, it is important to note that not all PUs are avoidable^7^. Limited information is available regarding the prevalence of PUs in long-term care institutions in Central Asia. Therefore, the aim of this study was to identify the prevalence rates and risk factors associated with PUs among residents of long-term care institutions in the Republic of Kazakhstan.

## Materials and methods

### Study design and setting

This prospective cross-sectional study was conducted between May 6 and 10, 2019. The study population consisted of residents of long-term care facilities, specifically hospices and nursing homes. The first step of the study involved creating a list of all long-term care medical institutions in Kazakhstan, which was based on information obtained from the Ministry of Health’s website^8^. Subsequently, on January 14, 2019, invitations to participate in the study were sent to 25 nursing homes and 9 hospices via institutional email. Among the contacted organizations, 9 institutions with varying bed capacities, ranging from 30 to 1495, responded to the invitation before March 14, 2019, and thus became participants in the study.

### Training and data collection

As the next step, an online training session was conducted by the principal investigator (Zh.Zh) for the designated nurses who were selected by the management to participate in the study as enumerators. The training sessions were held between April 8 and 10, 2019, during which the nurses received detailed instructions on how to complete the informed consent forms and utilize the study questionnaires, including training on how to recognize PU stages. Subsequently, the data collection phase commenced. During the data collection process, one institution decided to withdraw from the study without providing any explanation. Following the conclusion of the study, a quality check was conducted on the collected data, revealing that four institutions had collected data of suboptimal quality, with numerous missing values. Consequently, the records from these institutions were excluded from the final dataset. As a result, this study included data from two nursing homes and two hospices located in the cities of Astana, Almaty, and Semey.

### The instruments

The study utilized a collection of internationally recognized instruments to assess various aspects related to PU development. These instruments included the International Prevalence Measurement of Care Quality (Landelijke Prevalentiemeting Zorgkwaliteit, LPZ) questionnaire. Permission to employ the LPZ-International questionnaire^9^ was obtained from the LPZ project group at Maastricht University, the Netherlands (Protocol dated 20.10.2019). The LPZ-International questionnaire was developed through literature and expert opinion. Currently, it serves as a standardized registration system in the Netherlands for the annual assessment of PU prevalence. Besides the Netherlands, organizations in Switzerland, Austria, Turkey, and the United Kingdom also make use of the questionnaire. The LPZ questionnaire was used to record patients’ demographic data and pertinent characteristics related to PUs. These included age, gender, skin color, underlying diseases, length of hospital stay, recent surgical procedures within the past two weeks, duration of surgery, PU history over the past five years, history of bed rest, PU risk scale score utilizing the Braden scale^10^, and Care Dependency Score using the CDS^11^.

The Braden scale is a widely recognized and user-friendly instrument for assessing PU risk and is extensively employed in various countries. Its validity and reliability have been established through verification studies^9,12,13^. Comprising six subscales—sensory perception, moisture, activity, mobility, nutrition, friction and shear—the Braden scale employs interval scales for each subscale, with the exception of friction and shear, which employs three interval scales: problem, potential problem, and no apparent problem. A lower score on the scale indicates a higher likelihood of significant problems. The total score on the Braden scale ranges from 6 to 23^10^.

In addition to the Braden scale, the assessment of care dependency was conducted using the CDS^14,15^ to examine the association between dependency on nursing care and the occurrence of PUs. The CDS evaluates the extent of a patient’s physical and psychosocial care dependency. It comprises 15 items measuring various aspects of care dependency, including eating and drinking, continence, body posture, mobility, day and night patterns, dressing and undressing, body temperature, hygiene, avoidance of danger, communication, contact with others, sense of rules and values, daily activities, recreational activities, and learning ability. Each item employs a Likert-type scale with five categories, ranging from ‘1 = completely dependent’ to ‘5 = almost independent’. The total score on the CDS ranges from 15 to 75, with a lower score indicating a higher level of patient care dependency^16^. Also this tool assesses various specific characteristics of PUs, including PU categories, locations, the place where the PU developed (such as the current care unit, other units, other institutions, or home), duration of suffering from PUs, combination of PUs and moisture lesions, PU infections, and pain related to PUs. The PU categories are classified into four types: category I, referred to as ‘non-blanchable erythema’; category II, denoted as ‘partial thickness’; category III, defined as ‘full thickness skin loss’; and category IV, characterized as ‘full thickness tissue loss’. These categories are based on the classification system established by the European Pressure Ulcer Advisory Panel (EPUAP)^18^. Additionally, the questionnaire assessed the preventive measures implemented for the patients. PU prevention measures included repositioning, utilization of PU-redistributing devices, patient or family education, nutrition support, and skin moisturizing, among others.

Before initiating the study, a team of researchers (L.K., A.K., Zh.Zh) diligently undertook the translation of the LPZ-International Questionnaire into Kazakh and Russian languages. Subsequently, two independent researchers (Yu.S., G.Zh) carried out back word translations of the questionnaire into English. Through a rigorous process that encompassed rectifying deficiencies and integrating valuable feedback, the questionnaire underwent a trial phase involving a group of 32 individuals who were subsequently excluded from our study. The modifications and insights gathered during this testing phase were carefully evaluated and incorporated into the final version of the translated questionnaire.

### Definitions used

A pressure ulcer is specifically defined as “a localized injury to the skin and/or underlying tissue, typically occurring over a bony prominence, caused by pressure or a combination of pressure and shear forces”^17^.

A category I pressure ulcer is characterized by intact skin with non-blanchable redness in a localized area, typically occurring over a bony prominence. This area may exhibit characteristics such as pain, firmness, softness, and a different temperature (warmer or cooler) compared to the surrounding tissue. It is worth noting that detecting category I ulcers may pose challenges in individuals with darker skin tones. A category II is partial thickness loss of the dermis manifests as a shallow open ulcer with a red-pink wound bed, lacking slough. Alternatively, it may present as an intact or open/ruptured blister filled with serum. This type of ulcer appears as a shiny or dry shallow wound without slough. A category III the condition involves full-thickness tissue loss, where the subcutaneous fat may be discernible, but there is no exposure of bone, tendon, or muscle. The wound may exhibit undermining and tunneling. The depth of a category III pressure ulcer varies based on the specific anatomical location. The visibility or direct palpability of bone or tendon is not observed in such cases. A category IV is characterized by full-thickness tissue loss, wherein bone, tendon, or muscle is exposed. Slough or eschar may be present in certain areas of the wound bed. Undermining and tunneling are frequently observed characteristics. The depth of a category IV pressure ulcer varies depending on the anatomical location. Exposed bone or tendon is visibly apparent or can be directly palpated.

### Data collection

The four organizations participating in the study created a team of two nurses responsible for examining patients and collecting data. All the nurses were employed by these organizations. In the Semey hospice, the principal investigator (Zh.Zh), a certified nurse, and the assisting nurse collected the data. Prior to participating in the study, all the enrolled individuals underwent a preliminary briefing session that lasted 6 h. The training covered various areas, including the utilization of the LPZ-International questionnaire, the assessment of PU categorization and moisture damage, as well as the use of the CDS and Braden scale. To complete the patient demographics section of the questionnaire, nurses were permitted to extract information from patient registration cards. Subsequently, with the patient’s consent, a pair of nurses conducted a comprehensive examination of each patient, paying careful attention to the condition of the skin from head to toe, with a particular focus on bony prominences.

In instances where data collection was impeded by the patient’s cognitive impairment, family members or legal guardians were allowed to respond to all pertinent questions. Following the examination of each patient, the nurse was responsible for independently entering the obtained data into a specially developed online version of the LPZ questionnaire. The accuracy of data entry in the online questionnaires was verified by a group of researchers (L.K., A.K., A.A.).

### Statistical analysis

Data was analyzed using R 4.2.3 (R Foundation for Statistical Computing, Vienna, Austria). PU prevalence is the percentage of participants with at least one PU on the assessment day. It can be calculated including or excluding Category I PUs. If a participant has multiple PUs, only the highest grade is considered for calculating prevalence.

Descriptive statistics for continuous variables were reported as mean (standard deviation) and median (1st and 3rd quartiles). Categorical variables describing the characteristics of participants with PUs as well as the characteristics of their ulcers were presented with absolute and relative frequencies. To compare between groups, the Mann–Whitney U-test and Pearson’s chi-squared test were used for continuous and categorical variables, respectively. Univariate logistic regression was employed to estimate the association between candidate predictors and the outcome. Variables that showed a significant association with the outcome in the univariate analysis were included in a step-wise backward selection process based on the Akaike information criterion (AIC).

### Ethics approval and consent to participate

Ethical approval is obtained from the local Ethical Committee, Semey Medical University, Semey, Kazakhstan (Protocol #2, dated 25.10.2018) and the research was conducted in compliance with principles of the Declaration of Helsinki and the Guideline for Good Clinical Practice. All participants provided written informed consent.

## Results

At the time of data collection, four long-term care medical institutions had 18 care units with 1495 residing patients, 640 of whom agreed to participate, resulting in a response rate of 42.8% The reasons for non-participation included 455 patients who refused, 241 patients who were unavailable, 31 patients who were too ill or in a terminal condition, and 58 patients with unknown reasons for non-participation.

### PU prevalence

The overall prevalence of PUs categorized as I-IV was found to be 37% with a 95% confidence interval (CI) ranging from 33.4 to 40.8%. When excluding Category I PUs, the prevalence of PUs decreased to 35.6% [95% CI 32% to 39.4%].

Table 1 provides a comprehensive overview of the characteristics of patients presenting with PUs. The table reveals that a total of 237 participants had PUs, resulting in a combined total of 800 wounds. The majority of participants (22.8%) experienced PUs falling under category “Unstageable”. PUs belonging to Categories I, II, III, IV accounted for 3.8%, 17.3%, 16.5% and 20.7% respectively. This study identified 19% of PUs classified as “deep tissue injuries”. The principal sites exhibiting the presence of pressure ulcers (PUs) were the left trochanter (92 occurrences, accounting for 11.5% of cases), left ankle (90 occurrences, constituting 11.2% of cases), left heel (84 occurrences, representing 10.5% of cases), and right trochanter (80 occurrences, making up 10% of cases).

**Table 1:** PU characteristics of hospitalized Kazakhstani PU patients (n = 237).

Characteristics of PUs	Category I	Category II	Category III	Category IV	Unstageable	Deep tissue injuries
The highest PU category per subject (n = 237 subjects)	9 (3.8%)	41 (17.3%)	39 (16.5%)	49 (20.7%)	54(22.8%)	45 (19%)
Number of PU wounds per category (n = 800 wounds)	141 (17.6%)	212 (26.5%)	107 (13.4%)	138 (17.2%)	142 (17.8%)	60 (7.5%)
Locations of PU wounds (n = 800 wounds)						
Sacrum	10 (1.2%)	15 (1.9%)	16 (2%)	32 (4%)	4 (0.5%)	–
Left trochanter	3 (0.4%)	21 (2.6%)	5 (0.6%)	38 (4.8%)	3 (0.4%)	22 (2.8%)
Right trochanter	9 (1.1%)	30 (3.8%)	8 (1%)	–	15 (1.9%)	18 (2.2%)
Left ischium	17 (2.1%)	21 (2.6%)	11 (1.4%)	11 (1.4%)	1 (0.1%)	8 (1%)
Right ischium	9 (1.1%)	11 (1.4%)	1 (0.1%)	3 (0.4%)	2 (0.2%)	6 (0.8%)
Left heel	11 (1.4%)	22 (2.8%)	12 (1.5%)	17 (2.1%)	5 (0.6%)	17 (2.1%)
Right heel	14 (1.8%)	8 (1%)	4 (0.5%)	5 (0.6%)	8 (1%)	23 (2.9%)
Left ankle	26 (3.2%)	29 (3.6%)	4 (0.5%)	6 (0.8%)	5 (0.6%)	20 (2.5%)
Right ankle	7 (0.9%)	–	–	2 (0.2%)	5 (0.6%)	16 (2%)
Left elbow	10 (1.2%)	13 (1.6%)	44 (5.5%)	10 (1.2%)	–	–
Right elbow	12 (1.5%)	7 (0.9%)	–	1 (0.1%)	3 (0.4%)	7 (0.9%)
Left head	4 (0.5%)	17 (2.1%)	–	12 (1.5%)	–	1 (0.1%)
Other	2 (0.2%)	1 (0.1%)	–	1 (0.1%)	–	1 (0.1%)

### Characteristics of PU patients

Table 2 shows the demographic characteristics and prevalence of different disorders of non-PU participants and PU participants. Notable distinctions were observed between the groups in terms of factors such as age and medical conditions (including diabetes and other endocrine disorders, stroke and other nervous diseases, impairments of digestive and genitourinary systems, disorders of skin, connective tissue and muscles, malignancies, and end of life condition). The average age of PU patients (55.7 years, with a standard deviation of 11) was higher compared to non-PU patients (mean age of 53.4 years, with a standard deviation of 10.2). Among the PU patients, the majority had a stroke (39.2%), followed by genitourinary problems (18.6%), and cancer/neoplasms (15.6%).

**Table 2:** Basic characteristics and diseases/disorders of study participants.

Participant characteristics	Overall n = 640	Non-PU n = 403	PU n = 237	p-value
Sex				0.75
Male	398 (62.2%)	253 (62.8%)	145 (61.2%)	
Female	242 (37.8%)	150 (37.2%)	92 (38.8%)	
Age (years)	54.3 (10.6)	53.4 (10.2)	55.7 (11)	0.017
	52.7 (44.1‒64.5)	51.9 (43.3‒62.1)	53 (46.1‒65)	
End of life	4 (0.6%)	0 (0%)	4 (1.7%)	0.036
Diseases				
Diabetes mellitus	140 (21.9%)	127 (31.5%)	13 (5.5%)	< 0.001
Stroke	110 (17.2%)	17 (4.2%)	93 (39.2%)	< 0.001
Cancer/neoplasms	67 (10.5%)	30 (7.4%)	37 (15.6%)	0.002
Diseases of the skin and subcutaneous	66 (10.3%)	54 (13.4%)	12 (5.1%)	0.001
Diseases of the digestive system	44 (6.9%)	39 (9.7%)	5 (2.1%)	< 0.001
Diseases of the genitourinary system	44 (6.9%)	0 (0%)	44 (18.6%)	< 0.001
Spinal cord lesions/paraplegia	38 (5.9%)	17 (4.2%)	21 (8.9%)	0.026
Diseases of the nervous system (not spinal cord lesions/paraplegia)	29 (4.5%)	21 (5.2%)	8 (3.4%)	0.378
Diseases of the respiratory system	24 (3.8%)	23 (5.7%)	1 (0.4%)	0.001
Endocrine, nutritional, or metabolic diseases (not diabetes mellitus)	23 (3.6%)	23 (5.7%)	0 (0%)	< 0.001
Diseases of the musculoskeletal system and connective tissue	21 (3.3%)	19 (4.7%)	2 (0.8%)	0.015
Diseases of the circulatory system (not stroke)	17 (2.7%)	16 (4%)	1 (0.4%)	0.015
Diseases of the blood or blood-forming organs and disorders involving the immune mechanism	7 (1.1%)	7 (1.7%)	0 (0%)	0.100
Dementia	4 (0.6%)	4 (1%)	0 (0%)	0.308
Mental and behavioural disorders (not dementia or addiction)	3 (0.5%)	3 (0.7%)	0 (0%)	0.464
Diseases of the ear and mastoid process	3 (0.5%)	3 (0.7%)	0 (0%)	0.464

Table 3 outlines the notable distinctions observed between non-PU and PU patients concerning the presence of PU guidelines and expert committees, confusion experienced within the last 7 days, care dependency scale, and PU risk.

**Table 3:** PU management, medical history, care dependency scale, and PU risk of study participants.

Participant characteristics	Overall n = 640	Non-PU n = 403	PU n = 237	p-value
PU guideline	437 (68.3%)	288 (71.5%)	149 (62.9%)	0.03
PU expert committee	437 (68.3%)	288 (71.5%)	149 (62.9%)	0.03
Resident participation	618 (96.6%)	398 (98.8%)	220 (92.8%)	< 0.001
Surgery last 7 days	20 (3.1%)	10 (2.5%)	10 (4.2%)	0.325
Confusion last 7 days	64 (10%)	4 (1%)	60 (25.3%)	< 0.001
Aggressive last 7 days	3 (0.5%)	0 (0%)	3 (1.3%)	0.096
Care dependency scale, mean (SD)	2.9 (1.5)	2.9 (1.5)	2.8 (1.4)	0.087
Care dependency scale				0.024
Almost independent	350 (54.7%)	235 (58.3%)	115 (48.5%)	
A limited extent dependent	100 (15.6%)	55 (13.6%)	45 (19%)	
Partially dependent	22 (3.4%)	10 (2.5%)	12 (5.1%)	
A great extent dependent	95 (14.8%)	53 (13.2%)	42 (17.7%)	
Completely dependent	73 (11.4%)	50 (12.4%)	23 (9.7%)	
PU risk	419 (65.5%)	196 (48.6%)	223 (94.1%)	< 0.001

The mean PU risk score, as assessed using the Braden scale, exhibited a significant increase in patients with PUs when compared to non-PU patients (mean total score 3.2, SD 1.3 vs. mean total score 0.9, SD 1.2). Consequently, a higher proportion of PU patients fell into the high or very high-risk categories compared to non-PU patients. Furthermore, the mean total scores for all Braden scale items (including sensory perception, moisture, activity, mobility, nutrition, and friction and shear) were notably lower in PU patients as compared to the non-PU group (refer to Table 4).

**Table 4:** Total Braden score and Braden scale items of study participants.

Participant characteristics	Overall n = 640	Non-PU n = 403	PU n = 237	p-value
Braden scale, mean (SD)	1.7 (1.7)	0.9 (1.2)	3.2 (1.3)	< 0.001
Braden scale				< 0.001
No risk	221 (34.5%)	207 (51.4%)	14 (5.9%)	
Mild	143 (22.3%)	112 (27.8%)	31 (13.1%)	
Moderate	40 (6.2%)	37 (9.2%)	3 (1.3%)	
High	54 (8.4%)	12 (3%)	42 (17.7%)	
Very high	182 (28.4%)	35 (8.7%)	147 (62%)	
Sensory perception, mean (SD)	1.8 (1.1)	2.3 (0.8)	0.9 (1)	< 0.001
Sensory perception				< 0.001
No impairment	208 (32.5%)	193 (47.9%)	15 (6.3%)	
Slightly limited	224 (35%)	165 (40.9%)	59 (24.9%)	
Very limited	89 (13.9%)	27 (6.7%)	62 (26.2%)	
Completely limited	119 (18.6%)	18 (4.5%)	101 (42.6%)	
Nutrition, mean (SD)	1.4 (1.1)	1.8 (1.2)	0.8 (0.8)	< 0.001
Nutrition				< 0.001
Excellent	167 (26.1%)	154 (38.2%)	13 (5.5%)	
Adequate	94 (14.7%)	81 (20.1%)	13 (5.5%)	
Probably inadequate	207 (32.3%)	88 (21.8%)	119 (50.2%)	
Very poor	172 (26.9%)	80 (19.9%)	92 (38.8%)	
Activity, mean (SD)	1.5 (1.2)	2 (1.1)	0.6 (0.8)	< 0.001
Activity				< 0.001
Walks frequently	188 (29.4%)	178 (44.2%)	10 (4.2%)	
Walks occasionally	95 (14.8%)	78 (19.4%)	17 (7.2%)	
Chair fast	188 (29.4%)	106 (26.3%)	82 (34.6%)	
Bedfast	169 (26.4%)	41 (10.2%)	128 (54%)	
Mobility, mean (SD)	1.4 (1.2)	2 (1)	0.5 (0.8)	< 0.001
Mobility				< 0.001
No limitations	188 (29.4%)	178 (44.2%)	10 (4.2%)	
Slightly limited	98 (15.3%)	88 (21.8%)	10 (4.2%)	
Very limited	161 (25.2%)	99 (24.6%)	62 (26.2%)	
Completely immobile	193 (30.2%)	38 (9.4%)	155 (65.4%)	
Friction and shear, mean (SD)	0.9 (0.9)	1.2 (0.9)	0.3 (0.6)	< 0.001
Friction and shear				< 0.001
No apparent problem	233 (36.4%)	214 (53.1%)	19 (8%)	
Potential problem	84 (13.1%)	56 (13.9%)	28 (11.8%)	
Problem	323 (50.5%)	133 (33%)	190 (80.2%)	
Moisture, mean (SD)	2 (1.2)	2.5 (0.9)	1.2 (1.2)	< 0.001
Moisture				< 0.001
Rarely moist	344 (53.8%)	286 (71%)	58 (24.5%)	
Occasionally moist	95 (14.8%)	71 (17.6%)	24 (10.1%)	
Very moist	82 (12.8%)	19 (4.7%)	63 (26.6%)	
Constantly moist	119 (18.6%)	27 (6.7%)	92 (38.8%)	

Table 5 shows the results of multivariate analysis into risk factors related to the prevalence of PU. The resulting logistic regression models is characterized by Nagelkerke’s pseudo-R^2^ 0.88 and C-index (AUC) 0.98 [95% CI 0.97; 0.99]. The logistic regression analysis revealed that certain factors significantly increased the odds of presenting with PUs among residents of long-term care medical institutions. These factors included the history of stroke (OR 5.22; 95% CI 1.33–21.95), the diseases of digestive system (OR 10.01; 95% CI 1.35–79.65), presence of spinal cord lesions/paraplegia (OR 20.50; 95% CI 2.80–173.96), confusion reported within the last 7 days (OR 184.00; 95% CI 28.70–1379.00), and a limited extent dependency according to the CDS (OR 4.44; 95% CI 1.31; 16.1). Conversely, several factors were found to be significantly associated with decreased odds of having PUs. These factors included adherence to the PU prevention guidelines (OR 0.14; 95% CI 0.04–0.42), partial dependency (OR 0.08; 95% CI 0.01–0.55), and complete dependency (OR 0.01; 95% CI 0.00–0.04) according to the CDS.

**Table 5:** Risk factors associated with the prevalence of PUs.

Predictor	Odds ratio	95% CI	p-value	
Stroke	5.22	1.33; 21.95	0.019	
Diseases of the digestive system	10.01	1.35; 79.65	0.023	
Spinal cord lesions/paraplegia	20.50	2.80; 173.96	0.004	
PU guideline	0.14	0.04; 0.42	< 0.001	
Confusion last 7 days	184.00	28.70; 1379.00	< 0.001	
Care dependency scale				< 0.001
Limited extent dependent	4.44	1.31; 16.10	0.019	
Partially dependent	0.08	0.01; 0.55	0.018	
A great extent dependent	0.67	0.12; 3.56	0.649	
Completely dependent	0.01	0.00; 0.04	< 0.001	
Sensory perceptions				0.066
Lightly limited	1.33	0.40; 4.33	0.641	
Very limited	0.24	0.04; 1.59	0.135	
Completely limited	1.44	0.19; 12.10	0.730	
Activity				0.11
Walks occasionally	0.7	0.06; 6.14	0.765	
Chair fast	0.24	0.02; 2.16	0.228	
Bedfast	1.31	0.08; 17.52	0.844	

## Discussion

This study offers first insights into the prevalence and risk factors of PUs among the long-term care residents in Kazakhstan. The findings indicate that the overall prevalence of PUs, including Category I, was 37%, while the prevalence excluding Category I was 35.6%. Several factors strongly associated with PUs among patients of long-term care institutions were the history of stroke, the diseases of digestive system, presence of spinal cord lesions/paraplegia, confusion reported within the last 7 days, and a limited extent dependency according to the care dependency scale. Following the PU prevention guidelines and being partially or completely dependent, as measured by the care dependency scale, were linked to a notable reduction in the likelihood of developing pressure ulcers.

The prevalence of PUs among residents of long-term care medical facilities in Kazakhstan can be considered high. By conducting a meta-analysis, Li et al. reported a pooled global prevalence rate of 12.8% for pressure ulcers among a sample of 1,366,848 hospitalized adult patients, providing a basis for comparison^3^. The high prevalence of pressure ulcers in our study can be attributed to several factors. Firstly, the vast majority of the PU patients (223 of 237) in the included residential houses were found to be at risk of developing pressure ulcers, which contributes to the overall high prevalence. Additionally, variations in prevention and treatment practices, as well as differences in structural quality indicators across healthcare systems of different countries, may also play a role in the observed prevalence. For instance, PU prevalence of 26.7–46.2% with mean of 35.2% was reported for the patients with spinal cord injuries in the hospitals of developing countries^18^. For comparison, some studies demonstrated the prevalence rates of PUs in patients of 9.6% in Japan, 16% in Norway, and 24% in North America^19–21^. The majority of research on standards and quality of care in long-term care facilities focusing on the effectiveness of interventions are primarily carried out in European countries and the United States of America^22^. However, it is necessary to carry out additional research to explore the relationship between care quality and the prevalence of pressure ulcers in patients of Kazakhstani long-term care institutions.

Socio-economic vulnerability of patients could be a significant contributing factor to the higher prevalence of PUs among residents in public hospitals compared to private hospitals (9.9% in public hospitals versus 4.1% in private hospitals)^23^.

Our findings indicated that the majority of participants (43.9%) encountered PUs falling into Category II, whereas 13.1% of patients with PUs were categorized as “Category I” cases. This observation could potentially be attributed to inadequate management and/or self-care practices regarding the prevention and treatment of pressure ulcers among the participants in our study. Therefore, healthcare providers must proactively take measures to prevent the onset or deterioration of superficial stages. For PUs excluding Category I, the most prevalent locations were the sacrum, left trochanter, and left elbow. These anatomical sites might be exposed to the pressure during sleep. In addition, the PU participants had a history of stroke and spinal lesions/paraplegia, which affect greatly on mobility, activity, and sensory perception of patients.

Our study highlighted several risk factors linked to the occurrence of PUs among patients of long-term care medical institutions. According to our data, the history of stroke increased the odds of PU development 5 times. Among stroke patients hospitalized in a specialized hospital in Indonesia, 22% were diagnosed with PUs^24^. The recent study demonstrated that the pooled prevalence of pressure injuries in hospital studies and in patients’ homes without home healthcare services was 3.06%, while in nursing homes, it was 17.25%. Furthermore, the prevalence of pressure injuries among stroke patients was significantly higher after hospital discharge compared to during their hospital stay^25^. These findings emphasize the urgent need of high intensity post-stroke rehabilitation to prevent PUs. The presence of spinal cord lesions or paraplegia was strongly correlated with a higher prevalence of PUs among the participants in the study. This association can be readily explained by the fact that individuals with limited mobility or sensation, who are often confined to beds or wheelchairs, face the greatest risk of developing PUs^26^.

Diseases affecting the digestive system were identified as additional factors contributing to the development of PUs. This phenomenon can potentially be elucidated by the presence of nutritional disruptions that occur in instances of inflammation, degeneration, or other pathological conditions affecting various segments of the gastrointestinal tract. In its’ turn, inadequate nutrition and insufficient dietary intake are recognized as prominent risk factors contributing to the onset of pressure ulcers and compromised wound healing^27^. Maintaining an optimal nutritional status, which involves consuming suitable quantities of macronutrients and micronutrients, in addition to conducting timely nutritional assessments and risk stratification, plays a vital role in both the prevention of pressure ulcers and the promotion of their healing process^28^.

Another notable contributing factor to the heightened risk of PUs is the presence of confusion last 7 days. This fact can be primarily attributed to the adverse impact of limited cognitive abilities, impairing an individual’s capacity to effectively shift positions at regular intervals, identify sensations of discomfort, or seek timely assistance. Furthermore, mental confusion can impede the prompt detection and treatment of preexisting PUs, subsequently exacerbating the complications associated with such wounds. Moreover, the care demands of individuals experiencing confusion necessitate additional personnel and time allocation, presenting a challenge within the context of a typical healthcare facility^29^.

According to the findings of our study, adherence to PU prevention guidelines and higher dependency on care were identified as protective factors. Conversely, a limited degree of dependency was deemed a contributory factor to pressure ulcer development. This discrepancy could potentially be elucidated by the superior provision of nursing care specifically tailored for the most susceptible groups of individuals residing in long-term care facilities, particularly those who are immobilized. The research conducted by de Laat et al. presented evidence indicating that a previous history of PUs does not correlate with future responsible or compliant behavior in terms of health management among paraplegic patients^30^. Furthermore, the advantageous elements of interventions aimed at self-management support have demonstrated their efficacy, thereby providing valuable implications for healthcare professionals in terms of guiding their support for individuals at risk of PUs^31^.

Although this study employed a standardized questionnaire and strict adherence to the LPZ-International data collection procedure, it is important to acknowledge the limitations of this study. One of the main limitations of our study is the inclusion of only 4 out of 34 potential medical institutions for participation. As a consequence, our findings may not comprehensively represent the prevalence of PUs and their associated risk factors across all long-stay institutions in Kazakhstan. However, this study represents the inaugural investigation incorporating multiple hospitals situated in different cities across our country, rendering it a potentially invaluable resource for future, more extensive research endeavors. Secondly, because institutions agreed to support this study, random sampling was not feasible in this investigation. To effectively assess the burden of PU and quality of care at a national level, the implementation of a centralized measurement system, along with the support and guidance from the Ministry of Health, would significantly enhance efficiency. Finally, the institutions participating in this study did not collect the data on the same day as per the procedure outlined in the LPZ-International research. Nevertheless, we have tried to minimize the study time to meet the requirements as much as possible. In addition, two nurses simultaneously evaluated the data and examined the patient, lowering the possibility of a systematic error when calculating the Braden scale.

## Conclusions

To the best of our knowledge, this study represents the first investigation into the prevalence and risk factors related to pressure ulcers in the Republic of Kazakhstan. The comprehensive analysis of disease prevalence and the evaluation of care quality for patients with PUs are poised to contribute significantly to raising awareness among various stakeholders, including the general population, healthcare professionals, managers, and policymakers. The identification of key risk factors underscores the imperative to establish specialized training programs aimed at equipping medical personnel, relatives, and patients themselves with the necessary skills to provide optimal care for individuals affected by PUs. Furthermore, the treatment of underlying conditions, implementation of balanced nutritional strategies, provision of appropriate care, and effective prevention strategies collectively underscore the indispensability of a multidisciplinary approach to tackling this issue.

## Data Availability

The data and material are available from the corresponding author on request.

## Abbreviations

LPZ: Landelijke Prevalentiemeting Zorgkwaliteit
PU: Pressure ulcer
PUs: Pressure ulcers
CDS: Care Dependency Scale
DM: Diabetes mellitus
PAD: Peripheral arterial disease
COPD: Chronic obstructive pulmonary disease
EPUAP: European Pressure Ulcer Advisory Panel
AIC: Akaike information criterion
